# Epidemiologic Quantities for Monkeypox Virus Clade I from Historical Data with Implications for Current Outbreaks, Democratic Republic of the Congo

**DOI:** 10.3201/eid3010.240665

**Published:** 2024-10

**Authors:** Valentina Marziano, Giorgio Guzzetta, Ira Longini, Stefano Merler

**Affiliations:** Center for Health Emergencies, Fondazione Bruno Kessler, Trento, Italy (V. Marziano, G. Guzzetta, S. Merler);; University of Florida, Gainesville, Florida, USA (I. Longini)

**Keywords:** monkeypox virus, mpox, viruses, zoonoses, sexually transmitted infections, incubation period, serial interval, generation time, reproduction number, mpox clade I, Democratic Republic of the Congo

## Abstract

We used published data from outbreak investigations of monkeypox virus clade I in the Democratic Republic of the Congo to estimate the distributions of critical epidemiological parameters. We estimated a mean incubation period of 9.9 days (95% credible interval [CrI] 8.5–11.5 days) and a mean generation time of 17.2 days (95% CrI 14.1–20.9 days) or 11.3 days (95% CrI 9.4–14.0 days), depending on the considered dataset. Presymptomatic transmission was limited. Those estimates suggest generally slower transmission dynamics in clade I than in clade IIb. The time-varying reproduction number for clade I in the Democratic Republic of the Congo was estimated to be below the epidemic threshold in the first half of 2024. However, in the South Kivu Province, where the newly identified subclade Ib has been associated with sustained human-to-human transmission, we estimated an effective reproduction number above the epidemic threshold (95% CrI 0.96–1.27).

A large outbreak of monkeypox virus (MPXV) clade I (previously known as the Congo-Basin clade) infections has been ongoing in the Democratic Republic of the Congo (DRC) since the autumn of 2023 and had caused >20,000 cases and 1,000 deaths (mostly among children <15 years of age) across the country as of May 26, 2024 (*1*). Infections with MPXV clade I have a case-fatality ratio of up to 10% (*1*; L.K. Whittles et al., unpub. data, https://www.medrxiv.org/content/10.1101/2024.04.23.24306209v1), substantially more deadly than for infections with clade IIb (formerly West African clade), which has spread internationally since 2022, mainly through sexual contact among men who have sex with men (MSM) (*2*). 

The epidemiology of MPXV clade I seems to be evolving; until recently, it was mainly of zoonotic origin, and only sporadic human-to-human transmission occurred within households. Sexual transmission of MPXV clade I was observed during an outbreak investigation in Kwango Province, DRC, in March 2023 (*3*). A new subclade has been recently identified in Kamituga in the South Kivu Province, where extensive sexual transmission has been documented (*4*–*6*). Sustained human-to-human transmission, new routes of infections, and genetic evolution raise concerns about the potential risk for international spread, making estimating critical epidemiologic parameters specific to MPXV clade I urgent. In this study, we used previously published outbreak investigation data to provide novel estimates of probability distribution functions for epidemiologic parameters that are critical for monitoring and modeling the spread of MPXV clade I (K. Charniga et al., unpub. data, https://arxiv.org/abs/2405.08841).

## Methods

To estimate the incubation period, we fitted 3 families of distributions (i.e., Weibull, gamma, and log-normal, with a possible offset parameter) to data from 15 cases with known incubation period (i.e., the time elapsed between the infection episode and rash onset) reported in a published study (*7*). We estimated the distribution of the serial interval (i.e., the time elapsed between the symptom onset in an index case and in their secondary cases) on the basis of 2 different datasets and considering the same 3 families of distributions. We obtained the first dataset (dataset 1) by pooling together symptom onset dates for 32 infector–infectee transmission links from 2 household outbreaks in Sudan in 2005 (n = 13) (*8*) and in Central African Republic in 2021–2022 (n = 19) (*9*). The second dataset (dataset 2) consisted of 11 infector–infectee transmission links from a hospital-associated outbreak in the DRC in 2003 (*10*). In all 3 outbreaks, the chains of transmission were reconstructed through detailed epidemiologic investigations identifying the most likely infector. 

We estimated the generation time (i.e., the time elapsed between the date of exposure of a confirmed case and those of their secondary cases) by considering the same data on infector–infectee pairs used for the estimate of the serial interval, following a Bayesian approach previously applied to MPXV clade IIb (*11*,*12*). The generation time was assumed to be distributed either as a gamma, a Weibull, or a log-normal function with offset; parameters of the distributions and dates of exposure for all cases were estimated within a Markov chain Monte Carlo procedure. If the sampled date of exposure for the infectee was earlier than the date of symptom onset for the infector, we considered that an episode of presymptomatic transmission.

In sensitivity analyses, we accounted for potential double interval censoring caused by the discretization of data at a 1-day resolution for the incubation period and the serial interval, and we reestimated the generation time by not allowing for presymptomatic transmission (i.e., setting a null prior for exposure dates of the infectee occurring before the symptom onset date of the infector). 

We used estimates of the generation time to estimate reproduction numbers for MPXV clade I, using the timeseries of laboratory-confirmed mpox cases in the DRC during January 1–May 12, 2024 (*13*), and the timeseries of weekly hospitalized mpox case-patients (confirmed, probable, or suspected) in the Kamituga Health Zone, South Kivu Province, during late October 2023–April 21, 2024 (L.M. Masirika et al., unpub. data, https://www.medrxiv.org/content/10.1101/2024.05.10.24307057v1). The reproduction number, R_0_, is defined as the average number of secondary cases per infectious person and represents a critical parameter to assess the transmissibility of an infection; when R_0_<1, transmission is expected to fade out, whereas if R_0_>1, the epidemic has the potential for further spread. We computed the time-varying reproduction number, R_t_, representing transmissibility over time, and the effective reproduction number, R_eff_, representing an average value of transmissibility over the study period. We used the renewal equation and the computation of the exponential growth rate of the number of cases to provide estimates (*14*–*16*) (Appendix).

## Results

The best estimate for the incubation period was a Weibull distribution with an offset term of 4 days and mean of 9.9 days (95% credible interval [CrI] 8.5–11.5 days) (Figure, panel A). For the serial interval, we obtained as an optimal estimate a Weibull with an offset term of 1 day and mean of 17.5 days (95% CrI 14.1–21.5 days) for dataset 1 and a Weibull with offset 6 days and mean of 11.4 days (95% CrI 9.9–13.5 days) for dataset 2 (Figure, panel B).

**Figure:**
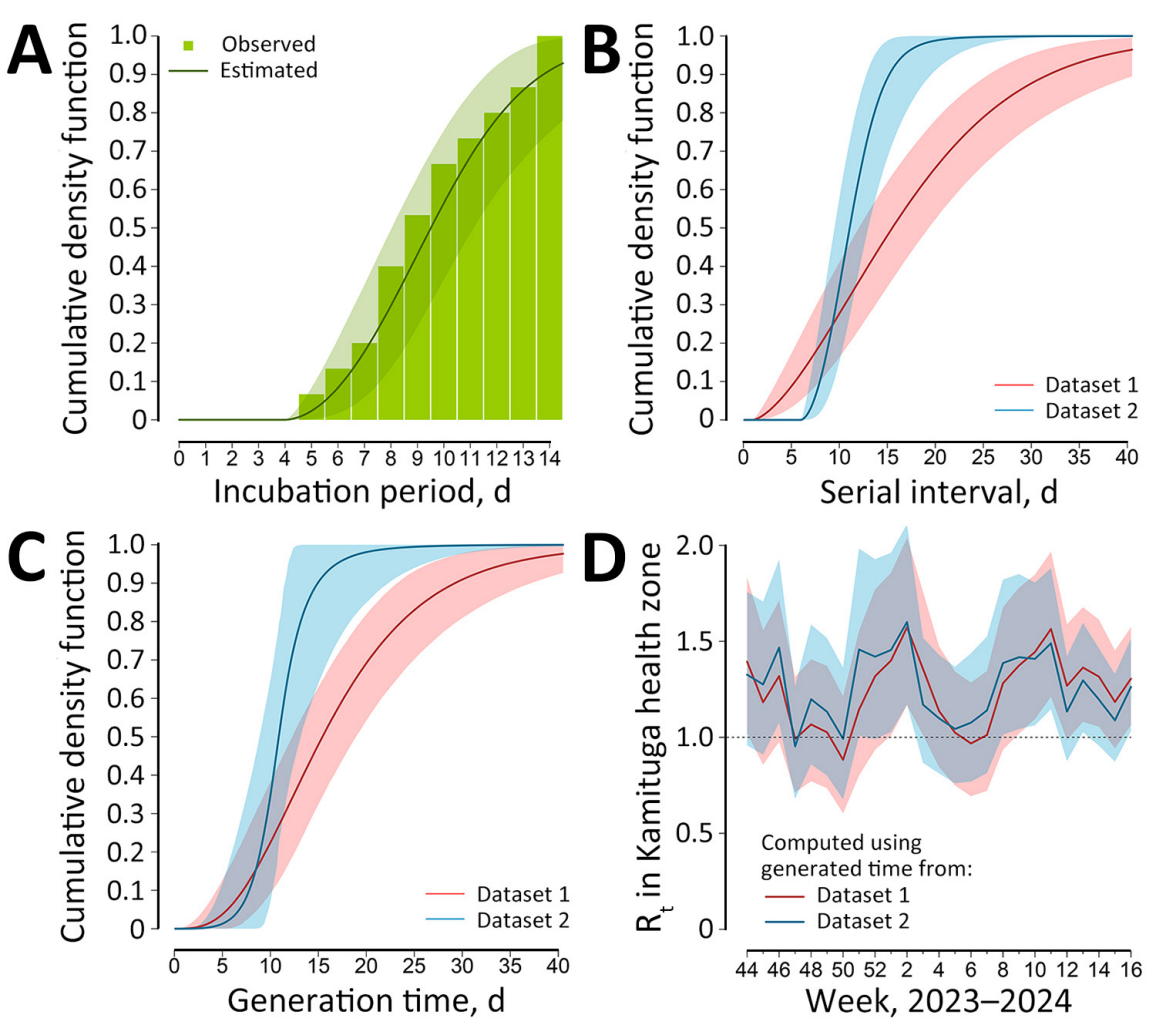
Estimates of key epidemiologic parameters for monkeypox virus clade I from historical data with implications for current outbreaks, Democratic Republic of the Congo. A) Cumulative density function of the incubation period, estimated from data on 15 cases reported in a previous study (*7*). B) Cumulative density function of the serial interval, estimated from data on 32 transmission links associated with household outbreaks (*8*,*9*), and on data on 11 transmission links associated with a hospital outbreak (*10*). C) Cumulative density function of the estimated generation time, based on the same data reported for the serial interval and on estimates of the incubation period. D) Estimates of R_t_ in the Kamituga Health Zone, obtained from the time-series of hospitalized cases (suspected, probable, and confirmed) (L.M. Masirika et al., unpub. data, https://www.medrxiv.org/content/10.1101/2024.05.10.24307057v1) and using the 2 estimates of the generation times. Lines indicate mean estimates; shaded areas indicate 95% credible intervals. R_t_, time-varying reproduction number.

The mean estimated generation time for the gamma distribution was 17.2 days (95% CrI 14.1–20.9 days) for dataset 1 and 11.3 days (95% CrI 9.4–14.0 days) for dataset 2 (Figure, panel C). For both considered datasets, presymptomatic transmission played a minor role (i.e., ≈20% of cases for dataset 1 and ≈17% of cases for dataset 2). For all parameters, variations were minimal when using the other 2 distribution families (Appendix). The distributions obtained from the sensitivity analyses accounting for potential double interval censoring and not allowing for presymptomatic transmission were practically overlapping with those from the baseline analysis (Appendix).

The mean R_t_ in the DRC was estimated to be under the epidemic threshold for most of 2024 (Appendix). However, the R_t_ estimated in Kamituga lay consistently above the epidemic threshold throughout the study period (Figure, panel D). Mean estimates for R_eff_ in Kamituga ranged from 1.08 to 1.18 (range of the 95% CrI 0.96–1.27), depending on the method and generation time considered. Additional results and full methods for the analysis are provided (Appendix).

## Discussion

The estimated mean incubation period of MPXV clade I (9.9 days, 95% CrI 8.5–11.5 days) was longer than most estimates reported for MPXV clade IIb in a review by the World Health Organization (*17*) (although some of the studies reported in the review might use a different definition of incubation period [e.g., from exposure to first symptoms rather than from exposure to rash as in our data]). Available data, although limited in sample size, point to a variability in serial intervals/generation times depending on the observation settings, with cases from household outbreaks having mean values ranging from 14 to 21 days (mean 17 days) and cases from an outbreak associated to hospital cases ranging from 9 to 14 days (mean 11 days). The transmission dynamics of MPXV clade I seem slower than clade IIb, for which mean estimates range from 5.6 to 9.4 days for serial intervals (*17*) and ≈12.5 days for generation times (*12*). We suggest a limited occurrence of presymptomatic transmission, on the basis of the estimated distribution of the infectious period and available data on serial intervals. This finding is again in contrast with MPXV clade IIb, for which substantial presymptomatic viral shedding and transmission has been demonstrated (*18*,*19*).

The estimated R_t_ for MPXV clade I in the DRC in 2024 has been hovering around values that are compatible with historical estimates of 0.75–0.86 (*20*–*22*), below the epidemic threshold of 1. However, the R_t_ estimated for the Kamituga Health Zone, South Kivu province, where a recently identified subclade has emerged (*5*), was above threshold; the mean value of R_eff_ was from 1.08 to 1.18 (95% CrI 0.96–1.27). This substantially higher value, compared with historical estimates, might be caused by possible viral adaptation to human transmission, as well as by higher local contact rates because of sexual transmission given the concentration of sex workers associated with the mining industry in the region (L.M. Masirika et al., unpub. data).

We acknowledge that parameter estimations could be affected by small sample sizes and by the limitations of primary studies, which makes extrapolating results to other transmission settings difficult (*7*–*10*). Further quantitative epidemiologic studies are warranted to improve the knowledge on this emerging pathogen. The precise estimation of R_t_ may be distorted by time-varying diagnostic delays, which shifts forward the epidemic curve and adds stochastic noise to the time series of cases. Estimation of R_t_ might also be affected by potential improvements in surveillance over time at the national level because of the outbreak declaration, by assumptions on the frequency of cases associated with zoonotic spillover, and by heterogeneous case definitions (laboratory-confirmed cases for the national-level curve vs. any hospitalized case for the Kamituga curve). Nonetheless, we do not expect the general conclusions to be affected by these potential biases. Estimates of the reproduction numbers might further be affected by the uncertainty of the distribution of the generation time; we note that, in principle, this distribution may be different for sexual transmission, which is currently the main route in South Kivu. However, estimates of reproduction numbers were relatively stable when using the 2 alternative generation time distributions estimated from heterogeneous datasets.

Those results show a distinct timing of MPXV clade I epidemiology compared with MPXV clade IIb, which suggests the need for investigations into the contribution of sexual transmission and associated serial intervals and generation times, and also provides useful parameters for monitoring and modeling the transmission dynamics of MPXV clade I. Finally, if not controlled with contact tracing and vaccination, this continued relatively low-level but persistent transmission of MPXV clade I in the DRC could lead to further evolution of the virus toward higher person-to-person transmissibility and further spread beyond the current geographic focus of transmission (*23*).

Appendix Additional information about epidemiologic quantities for monkeypox virus clade I from historical data with implications for current outbreaks, Democratic Republic of the Congo.

## References

[1] World Health Organization. Mpox—Democratic Republic of the Congo [cited 2024 Jun 25]. https://www.who.int/emergencies/disease-outbreak-news/item/2024-DON522

[2] World Health Organization. Mpox (monkeypox) outbreak 2022 [cited 2024 Jun 25]. https://www.who.int/emergencies/situations/monkeypox-oubreak-2022

[3] Kibungu EM, Vakaniaki EH, Kinganda-Lusamaki E, Kalonji-Mukendi T, Pukuta E, Hoff NA, et al.; International Mpox Research Consortium. Clade I–associated mpox cases associated with sexual contact, the Democratic Republic of the Congo. Emerg Infect Dis. 2024;30:172–6. 10.3201/eid3001.231164 38019211 PMC10756366

[4] Katoto PD, Muttamba W, Bahizire E, Malembaka EB, Bosa HK, Kazadi DM, et al. Shifting transmission patterns of human mpox in South Kivu, DR Congo. Lancet Infect Dis. 2024;24:e354–5. 10.1016/S1473-3099(24)00287-1 38703785

[5] Vakaniaki EH, Kacita C, Kinganda-Lusamaki E, O’Toole Á, Wawina-Bokalanga T, Mukadi-Bamuleka D, et al. Sustained human outbreak of a new MPXV clade I lineage in eastern Democratic Republic of the Congo. Nat Med. 2024; Epub ahead of print. 10.1038/s41591-024-03130-3 38871006 PMC11485229

[6] Masirika LM, Udahemuka JC, Schuele L, Ndishimye P, Otani S, Mbiribindi JB, et al. Ongoing mpox outbreak in Kamituga, South Kivu province, associated with monkeypox virus of a novel Clade I sub-lineage, Democratic Republic of the Congo, 2024. Euro Surveill. 2024;29:2400106. 10.2807/1560-7917.ES.2024.29.11.2400106 38487886 PMC10941309

[7] Nolen LD, Osadebe L, Katomba J, Likofata J, Mukadi D, Monroe B, et al. Extended human-to-human transmission during a monkeypox outbreak in the Democratic Republic of the Congo. Emerg Infect Dis. 2016;22:1014–21. 10.3201/eid2206.150579 27191380 PMC4880088

[8] Formenty P, Muntasir MO, Damon I, Chowdhary V, Opoka ML, Monimart C, et al. Human monkeypox outbreak caused by novel virus belonging to Congo Basin clade, Sudan, 2005. Emerg Infect Dis. 2010;16:1539–45. 10.3201/eid1610.100713 20875278 PMC3294404

[9] Besombes C, Mbrenga F, Malaka C, Gonofio E, Schaeffer L, Konamna X, et al. Investigation of a mpox outbreak in Central African Republic, 2021-2022. One Health. 2023;16:100523. 10.1016/j.onehlt.2023.100523 36950196 PMC9988319

[10] Learned LA, Reynolds MG, Wassa DW, Li Y, Olson VA, Karem K, et al. Extended interhuman transmission of monkeypox in a hospital community in the Republic of the Congo, 2003. Am J Trop Med Hyg. 2005;73:428–34. 10.4269/ajtmh.2005.73.428 16103616

[11] Miura F, van Ewijk CE, Backer JA, Xiridou M, Franz E, Op de Coul E, et al. Estimated incubation period for monkeypox cases confirmed in the Netherlands, May 2022. Euro Surveill. 2022;27:2200448. 10.2807/1560-7917.ES.2022.27.24.2200448 35713026 PMC9205160

[12] Guzzetta G, Mammone A, Ferraro F, Caraglia A, Rapiti A, Marziano V, et al. Early estimates of monkeypox incubation period, generation time, and reproduction number, Italy, May–June 2022. Emerg Infect Dis. 2022;28:2078–81. 10.3201/eid2810.221126 35994726 PMC9514338

[13] Monkeypox in the Democratic Republic of the Congo: epidemiological situation report sitrep no. 014 (06–12 May 2024) [in French] [cited 2024 Jun 25]. https://reliefweb.int/report/democratic-republic-congo/la-variole-simienne-monkeypox-en-republique-democratique-du-congo-rapport-de-la-situation-epidemiologique-sitrep-no014-06-12-mai-2024

[14] Cori A, Ferguson NM, Fraser C, Cauchemez S. A new framework and software to estimate time-varying reproduction numbers during epidemics. Am J Epidemiol. 2013;178:1505–12. 10.1093/aje/kwt133 24043437 PMC3816335

[15] Thompson RN, Stockwin JE, van Gaalen RD, Polonsky JA, Kamvar ZN, Demarsh PA, et al. Improved inference of time-varying reproduction numbers during infectious disease outbreaks. Epidemics. 2019;29:100356. 10.1016/j.epidem.2019.100356 31624039 PMC7105007

[16] Wallinga J, Lipsitch M. How generation intervals shape the relationship between growth rates and reproductive numbers. Proc Biol Sci. 2007;274:599–604. 10.1098/rspb.2006.3754 17476782 PMC1766383

[17] World Health Organization. 2022–24 mpox (monkeypox) outbreak: global trends. Literature summary & epidemic parameters [cited 2024 Jun 25]. https://worldhealthorg.shinyapps.io/mpx_global/#6_Literature_summary__epidemic_parameters

[18] Brosius I, Van Dijck C, Coppens J, Vandenhove L, Bangwen E, Vanroye F, et al.; ITM MPOX Study Group. Presymptomatic viral shedding in high-risk mpox contacts: A prospective cohort study. J Med Virol. 2023;95:e28769. 10.1002/jmv.28769 37212312

[19] Miura F, Backer JA, van Rijckevorsel G, Bavalia R, Raven S, Petrignani M, et al.; Dutch Mpox Response Team. Time scales of human mpox transmission in the Netherlands. J Infect Dis. 2024;229:800–4. 10.1093/infdis/jiad091 37014716 PMC10938196

[20] Fine PEM, Jezek Z, Grab B, Dixon H. The transmission potential of monkeypox virus in human populations. Int J Epidemiol. 1988;17:643–50. 10.1093/ije/17.3.643 2850277

[21] Sun YQ, Chen JJ, Liu MC, Zhang YY, Wang T, Che TL, et al. Mapping global zoonotic niche and interregional transmission risk of monkeypox: a retrospective observational study. Global Health. 2023;19:58. 10.1186/s12992-023-00959-0 37592305 PMC10436417

[22] Charniga K, McCollum AM, Hughes CM, Monroe B, Kabamba J, Lushima RS, et al. Updating reproduction number estimates for mpox in the Democratic Republic of Congo using surveillance data. Am J Trop Med Hyg. 2024;110:561–8. 10.4269/ajtmh.23-0215 38320310 PMC10919191

[23] Johnson PLF, Bergstrom CT, Regoes RR, Longini IM, Halloran ME, Antia R. Evolutionary consequences of delaying intervention for monkeypox. Lancet. 2022;400:1191–3. 10.1016/S0140-6736(22)01789-5 36152668 PMC9534010

