# Supplementary material for: Epidemiologic Quantities for Monkeypox Virus Clade I from Historical Data with Implications for Current Outbreaks, Democratic Republic of the Congo
Source: Emerg Infect Dis. 2024 Oct;30(10):2042–6. doi: 10.3201/eid3010.240665 (PMC11431919; doi:10.3201/eid3010.240665)
Supplement: Appendix — Additional information about epidemiologic quantities for monkeypox virus clade I from historical data with implications for current outbreaks, Democratic Republic of the Congo. [file 24-0665-Techapp-s1.pdf]

Article DOI: https://doi.org/10.3201/eid3010.240665

EID cannot ensure accessibility for supplementary materials supplied by authors. Readers who have difficulty accessing supplementary content should contact the authors for assistance.

Epidemiologic Quantities for Monkeypox Virus Clade I from Historical Data with Implications for Current Outbreaks, Democratic Republic of the Congo

Appendix

Incubation period

To estimate the distribution of the incubation period, we considered observations from 15 confirmed cases with known dates of exposure and symptom onset identified in (*1*) and reported in Appendix Table 1. We fitted three families of distributions (a gamma, a Weibull and a log-normal) assuming an offset of 4 days and using maximum likelihood estimation (MLE). The assumed value for the offset was based on the minimum observed incubation period of 5 days (Appendix Table 1). A Weibull distribution was selected as baseline, based on the minimum value of the Bayesian information Criterion (BIC) score (Appendix Table 2 and Appendix Figure 1). The posterior distributions of the shape (k) and scale parameters (λ) of the Weibull distribution were then estimated with a Monte Carlo Markov Chain (MCMC) procedure and Metropolis-Hastings sampling (*2*). Convergence of MCMC trace plots was evaluated visually (see Appendix Figure 2). The MCMC procedure was run also for the other two families of distributions (gamma and log-normal). Posterior distributions of all estimated parameters are reported in Appendix Table 3. The 2.5%, 25%, 50%, 75%, 97.5% quantiles as well as the mean and standard deviation for each fitted distribution are reported in Appendix Table 4.

Because incubation periods are provided in integer days, data may be interpreted as double-censored, since a patient could have experienced the event of interest (e.g., symptom

onset) at any time between 12:00 a.m. and 11:59 p.m. on a particular day. Therefore, we re-fitted the distributions after accounting for double-censoring, using the R package coarseDataTools version 0.5.1 (http://cran.r-project.org/web/packages/coarseDataTools/index.html), obtaining almost identical results (Appendix Tables 5, 6).

Appendix Table 1. Incubation periods (days) for 15 mpox cases with known dates of exposure and symptom onset (*1*).

ID case patient	1	2	3	4	5	6	7	8	9	10	11	12	13	14	15
Incubation periods (days)	5	6	7	8	8	8	9	9	10	10	11	12	13	14	14

Appendix Table 2. BIC score as obtained from MLE fit of the three families of distributions against data reported in Appendix Table 1*.

Distribution	BIC
Gamma	77.9
Weibull	**76.8**
Log-normal	80.2

*The best-fitting value of the three scores is highlighted in bold.

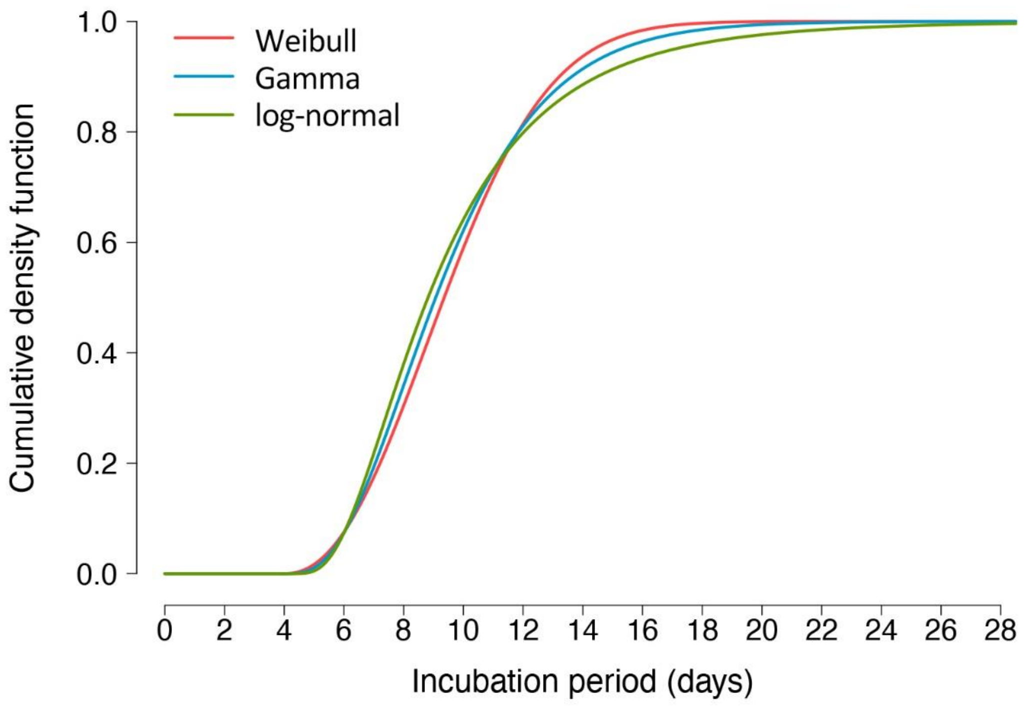

Appendix Figure 1. Incubation period of monkeypox virus (MPXV) clade I. Comparison between the cumulative probability distribution functions of the incubation period obtained from MLE for the three families of distributions.

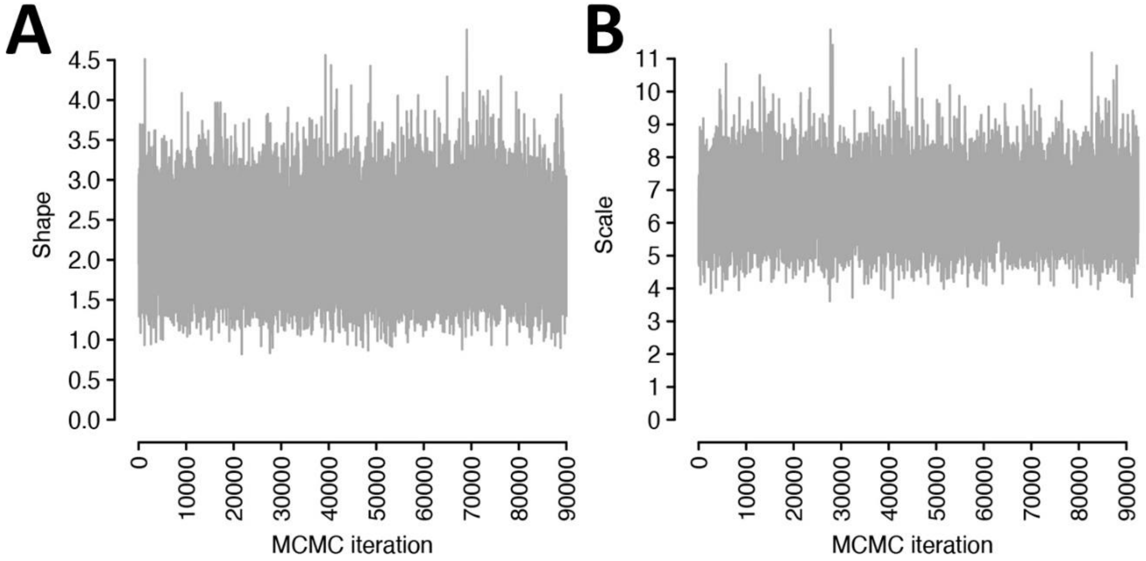

Appendix Figure 2. MCMC trace plots for the parameters of the Weibull distribution of the incubation period. A) Shape parameter. B) Scale parameter.

Appendix Table 3. Estimated parameters for the Weibull, gamma and log-normal distributions of the incubation period*.

Parameter	Weibull	gamma	log-normal
Offset (days)	4 (fixed)	4 (fixed)	4 (fixed)
Parameter 1: mean (95% CI)	Shape: 2.22 (1.38–3.22)	Shape: 3.21 (1.28–6.06)	Mean: 1.57 (1.21–1.94)
Parameter 2: mean (95% CI)	Scale: 6.56 (5.01–8.44)	Scale: 2.18 (0.91–5.58)	SD: 0.69 (0.47–1.04)

*Cells corresponding to the baseline distribution are highlighted in yellow. CI, **credible** interval; SD, standard deviation.

Appendix Table 4. Summary statistics for different estimates of the incubation period*

Incubation period estimate (days)	Percentile						
	2.5%	25%	50%	75%	97.5%	mean	SD
Weibull: mean (95% CI)	5.2 (4.4–6.3)	7.7 (6.3–9.1)	9.5 (8.0–11.1)	11.7 (10.0–14.0)	16.2 (13.3–22.1)	9.8 (8.5–11.5)	2.9 (2–4.6)
Gamma: mean (95% CI)	5.2 (4.3–6.2)	7.4 (6–8.7)	9.2 (7.7–11.1)	11.8 (9.7–15.3)	18.6 (13.9–28.9)	9.9 (8.3–12.4)	3.5 (2.1–6.5)
lognormal: mean (95% CI)	5.3 (4.6–6.1)	7.1 (5.9–8.3)	8.9 (7.4–10.9)	11.9 (9.4–16.3)	24.0 (15–46.1)	10.3 (8.3–14.2)	5.3 (2.5–13.0)

*For each estimate, and for each accepted sample of the joint posterior parameter distribution, we computed the 2.5%, 25%, 50%, 75%, 97.5% quantiles as well as the mean and standard deviation (SD); the table reports the mean values and 95% CI of each quantity computed across the samples of the joint posterior distribution. Cells corresponding to the baseline distribution are highlighted in yellow.

Appendix Table 5. Estimated parameters for the Weibull, gamma and log-normal distributions of the incubation period after accounting for double censoring interval due to the discretization of dates.

Parameter	Weibull	gamma	log-normal
Offset (days)	4 (fixed)	4 (fixed)	4 (fixed)
Parameter 1: mean (95% CI)	Shape: 2.08 (1.31–3.18)	Shape: 3.54 (1.51–7.18)	Mean: 1.58 (1.25–1.89)
Parameter 2: mean (95% CI)	Scale: 6.24 (4.63–7.92)	Scale: 1.55 (0.76–3.88)	SD: 0.60 (0.41–0.92)

Appendix Table 6. Summary statistics for different estimates of the incubation period after accounting for double censoring interval due to the discretization of dates*.

Incubation period estimate (days)	Percentile				
	2.5%	25%	50%	75%	97.5%
Weibull: mean (95% CI)	4.1 (4.3–6.1)	7.4 (5.9–8.8)	9.2 (7.6–10.8)	11.2 (9.7 −13.7)	15.8 (13.1–21.3)
gamma: mean (95% CI)	5.3 (4.3–6.4)	7.4 (6.0–8.6)	9.0 (7.6–10.7)	11.1 (9.5–13.7)	16.6 (13.4- 23.9)
lognormal: mean (95% CI)	5.5 (4.7–6.4)	7.2 (6.0–8.5)	8.9 (7.5–10.8)	11.4 (9.3–15.6)	19.3 (14.1- 35.3)

*For each estimate, and for each accepted sample of the joint posterior parameter distribution, we computed the 2.5%, 25%, 50%, 75%, 97.5%; the table reports the mean values and 95% CI of each quantity computed across the samples of the joint posterior distribution.

Serial interval

The serial interval is defined as the difference between the date of symptom onset of an infector and those of their infectees. We estimated the serial interval distribution using two different sets of infector-infectee pairs for which the dates of symptom onset were known. The first dataset consists of 32 pairs obtained by pooling together data from two household outbreaks in Sudan, 2005 (*3*) (n = 13) and in Central African Republic, 2021–2022 (*4*) (n = 19) (Appendix Table 7); the second dataset consists of 11 pairs from a hospital-associated outbreak in the Republic of the Congo, 2003 (*5*) (Appendix Table 8).

Appendix Table 7. Information on infector-infectee pairs from dataset 1.

ID pair	ID infector	ID infected	Symptom onset infector	Symptom onset infected	Observed serial interval	Source
1	B1	B2	06/11/2021	24/11/2021	18	Besombes et al. (*4*)
2	B1	B3	06/11/2021	27/11/2021	21	Besombes et al. (*4*)
3	B1	B4	06/11/2021	30/11/2021	24	Besombes et al. (*4*)
4	B1	B5	06/11/2021	08/11/2021	2	Besombes et al. (*4*)
5	B6	B7	07/11/2021	02/12/2021	25	Besombes et al. (*4*)
6	B6	B8	07/11/2021	06/12/2021	29	Besombes et al. (*4*)
7	B6	B9	07/11/2021	13/12/2021	36	Besombes et al. (*4*)
8	B4	B10	30/11/2021	02/12/2021	2	Besombes et al. (*4*)
9	B10	B11	02/12/2021	04/01/2022	33	Besombes et al. (*4*)
10	B7	B12	02/12/2021	08/12/2021	6	Besombes et al. (*4*)
11	B9	B13	13/12/2021	16/12/2021	3	Besombes et al. (*4*)
12	B9	B14	13/12/2021	19/12/2021	6	Besombes et al. (*4*)
13	B12	B15	08/12/2021	20/12/2021	12	Besombes et al. (*4*)
14	B12	B16	08/12/2021	23/12/2021	15	Besombes et al. (*4*)
15	B12	B17	08/12/2021	28/12/2021	20	Besombes et al. (*4*)
16	B12	B18	08/12/2021	01/01/2022	24	Besombes et al. (*4*)
17	B12	B19	08/12/2021	04/01/2022	27	Besombes et al. (*4*)
18	B12	B20	08/12/2021	12/01/2022	35	Besombes et al. (*4*)
19	B12	B21	08/12/2021	12/01/2022	35	Besombes et al. (*4*)
20	F1	F2	19/09/2005	30/09/2005	11	Formenty et al. (*3*)
21	F2	F3	30/09/2005	08/10/2005	8	Formenty et al. (*3*)
22	F2	F4	30/09/2005	08/10/2005	8	Formenty et al. (*3*)
23	F2	F5	30/09/2005	16/10/2005	16	Formenty et al. (*3*)
24	F2	F6	30/09/2005	16/10/2005	16	Formenty et al. (*3*)
25	F5	F7	16/10/2005	30/10/2005	14	Formenty et al. (*3*)
26	F5	F8	16/10/2005	03/11/2005	18	Formenty et al. (*3*)
27	F6	F9	16/10/2005	30/10/2005	14	Formenty et al. (*3*)
28	F10	F11	27/10/2005	05/11/2005	9	Formenty et al. (*3*)
29	F12	F13	01/12/2005	15/12/2005	14	Formenty et al. (*3*)
30	F12	F14	01/12/2005	15/12/2005	14	Formenty et al. (*3*)
31	F15	F16	24/10/2005	08/11/2005	15	Formenty et al. (*3*)
32	F15	F17	24/10/2005	08/11/2005	15	Formenty et al. (*3*)

Appendix Table 8. Information on infector-infectee pairs from dataset 2.

ID pair	ID infector	ID infected	Symptom onset infector	Symptom onset infected	Serial interval	Source
1	L1	L2	15/04/2003	28/04/2003	13	Learned et al. (*5*)
2	L2	L3	28/04/2003	08/05/2003	10	Learned et al. (*5*)
3	L3	L4	08/05/2003	18/05/2003	10	Learned et al. (*5*)
4	L3	L5	08/05/2003	18/05/2003	10	Learned et al. (*5*)
5	L4/L5	L6	18/05/2003	28/05/2003	10	Learned et al. (*5*)
6	L4/L5	L7	18/05/2003	30/05/2003	12	Learned et al. (*5*)
7	L6	L8	28/05/2003	05/06/2003	8	Learned et al. (*5*)
8	L6	L9	28/05/2003	05/06/2003	8	Learned et al. (*5*)
9	L7	L10	30/05/2003	10/06/2003	11	Learned et al. (*5*)
10	L10	L11	10/06/2003	23/06/2003	13	Learned et al. (*5*)
11	L8	L12	05/06/2003	22/06/2003	17	Learned et al. (*5*)

We used MLE to fit the same three families of distributions (gamma, Weibull and log-normal) to the observed serial intervals, assuming an offset of 1 day for dataset 1 and of 6 days for dataset 2, based on the minimum observed serial intervals (BIC scores reported in Appendix Table 9). For dataset 1, the best-fitting distribution was the Weibull, while for dataset 2 the log-normal was marginally better than the gamma and Weibull. Given the small differences among the distributions reported in Appendix Table 9 and shown in Appendix Figure 3, to maintain uniformity we selected as a baseline the Weibull distribution for dataset 2 as well. For both datasets, we estimated the posterior distributions of the shape (k) and scale parameters (λ) of the Weibull distribution, based on MCMC and Metropolis-Hastings sampling (*2*). Convergence of MCMC trace plots was evaluated visually (see Appendix Figures 5 and 6). The posterior distributions of the serial interval obtained for dataset 1 and dataset 2, respectively, are shown in Appendix Figure 4. The MCMC procedure was run also for the other two families of distributions (gamma and log-normal). Posterior distributions of all estimated parameters are reported in Appendix Table 10. The 2.5%, 25%, 50%, 75%, 97.5% quantiles as well as the mean and standard deviation for each fitted distribution are reported in Appendix Table 11. Again, we re-fitted the distributions after accounting for double-censoring, using the same R package coarseDataTools version 0.5.1 as above, and obtaining again almost identical results (Appendix Tables 12, 13).

Appendix Table 9. BIC score obtained from the MLE fit of the three families of distributions to serial interval data from the household and hospital outbreaks*.

Distribution	BIC	
	**Dataset 1 (n = 32)**	**Dataset 2 (n = 11)**
Gamma	241.5	53.64
Weibull	**239.2**	54.38
Log-normal	250.5	**53.61**

*The best-fitting values are highlighted in bold.

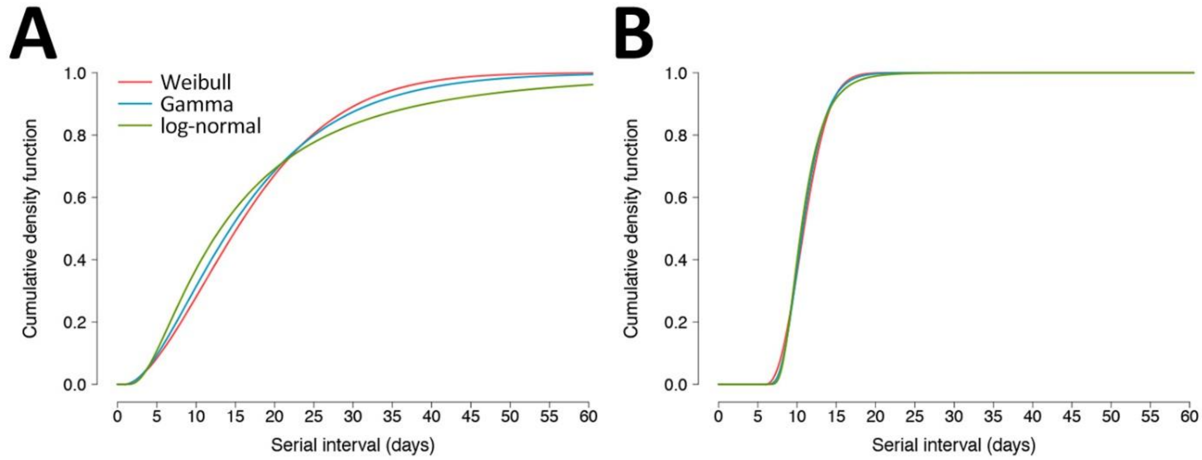

Appendix Figure 3. Comparison between the probability distribution functions of the serial interval obtained from MLE for the three families of distributions. A) dataset 1; B) dataset 2.

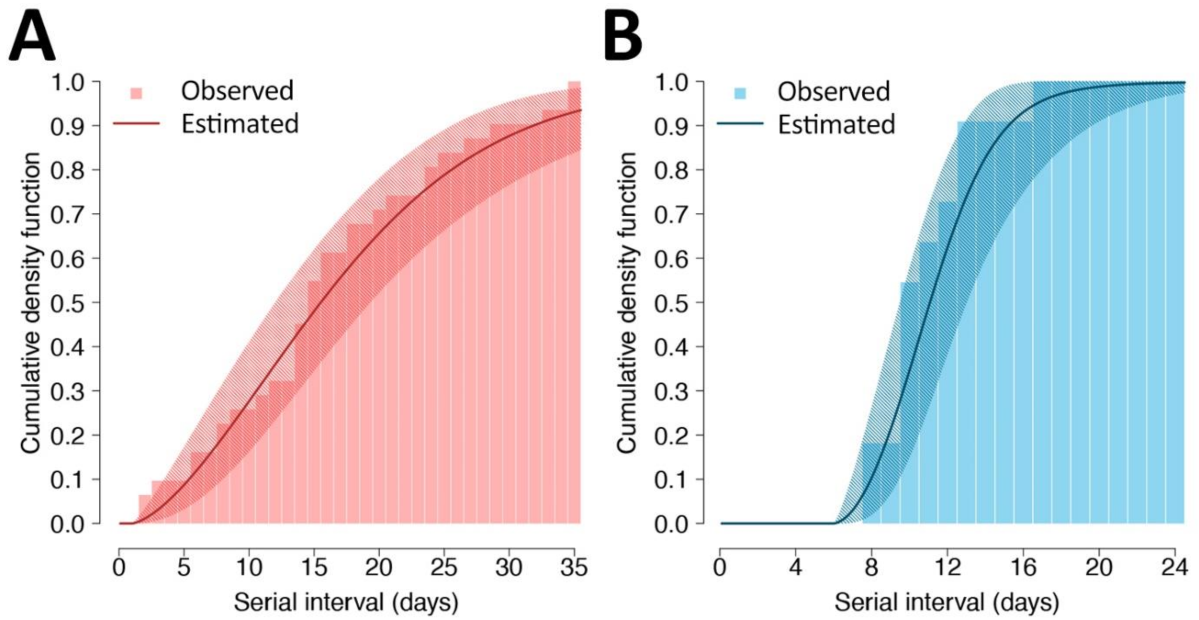

Appendix Figure 4. Serial interval of MPXV clade I. A) cumulative density function of the serial interval, estimated from dataset 1, consisting of 32 infector-infectee pairs reported in the literature in two household outbreaks (*3*,*4*). B) cumulative density function of the serial interval, estimated from dataset 2, consisting of 11 infector-infectee pairs reported in the literature in a hospital outbreak (*5*).

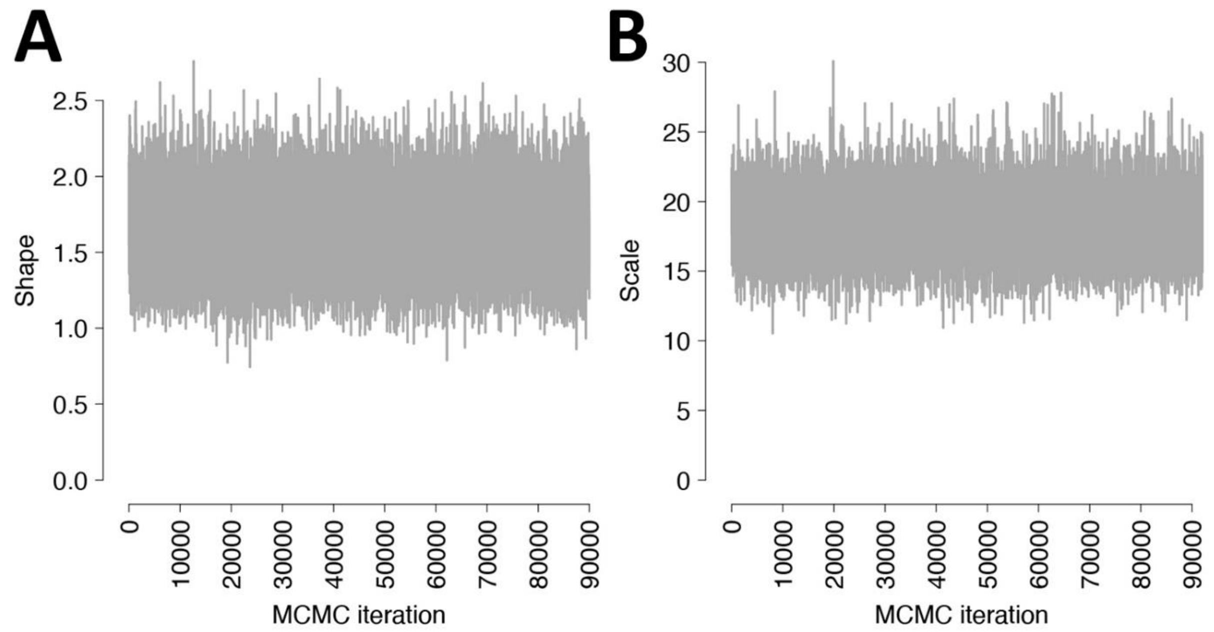

Appendix Figure 5. MCMC trace plots for the parameters of the Weibull distribution of the serial interval estimated from dataset 1. A) Shape parameter. B) Scale parameter.

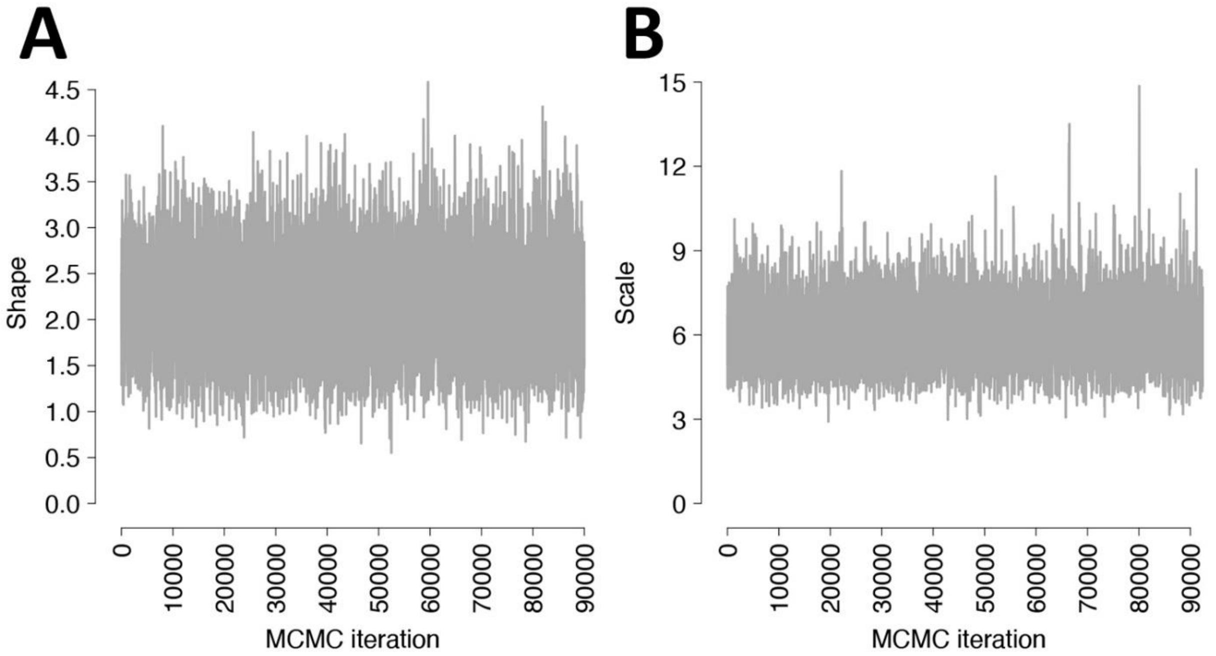

Appendix Figure 6. MCMC trace plots for the parameters of the Weibull distribution of the serial interval estimated from dataset 2. A) Shape parameter. B) Scale parameter.

Appendix Table 10. Estimated parameters for the Weibull, gamma and log-normal distributions of the serial interval for the two considered datasets*.

Distribution	Dataset 1<br>Weibull	gamma	log-normal	Dataset 2<br>Weibull	gamma	log-normal
Offset (days)	1 (fixed)	1 (fixed)	1 (fixed)	6 (fixed)	6 (fixed)	6 (fixed)
Parameter 1: mean (95% CI)	Shape: 1.63 (1.20–2.12)	Shape: 1.91 (1.15–2.88)	Mean: 2.50 (2.16–2.83)	Shape: 2.17 (1.29–3.19)	Shape: 4.15 (1.48–8.11)	Mean: 1.51 (1.14–1.88)
Parameter 2: mean (95% CI)	Scale: 18.29 (14.35–22.76	Scale: 9.21 (5.38–15.72)	SD: 0.95 (0.74–1.23)	Scale: 6.10 (4.38–8.38)	Scale: 1.59 (0.62–4.35)	SD: 0.6 (0.37–1.01)

*Cells corresponding to the baseline distribution are highlighted in yellow.

Appendix Table 11. Summary statistics for different estimates of the serial interval*.

	Percentile						
Dataset 1	2.5%	25%	50%	75%	97.5%	mean	SD
Serial interval – Weibull (days): mean (95% CI)	3.0 (1.8–4.5)	9.5 (6.6–12.4)	15.6 (12.0–19.3)	23.4 (18.9–29.1)	42.6 (33.1–58.4)	17.5 (14.1–21.5)	10.5 (7.8–15.1)
Serial interval –gamma (days): mean (95% CI)	2.8 (1.6–4.4)	8.6 (6.0–11.4)	14.7 (11.2–18.7)	23.4 (18.3–30.4)	48.9 (36.3–68.3)	17.6 (13.8–22.6)	12.3 (8.7–17.8)
Serial interval – lognormal (days): mean (95% CI)	3.0 (2.0–4.2)	7.5 (5.3–10.1)	13.3 (9.7–18)	24.5 (17.3–35.5)	83.3 (47.9–153.2)	20.7 (14.3–31.5)	25.1 (12.6–53.6)
Dataset 2							
Serial interval – Weibull (days): mean (95% CI)	7.1 (6.3–8.1)	9.4 (7.9–10.9)	11.1 (9.5–13)	13.2 (11.3–16.0)	17.6 (14.4–24.5)	11.4 (9.9–13.5)	2.8 (1.8–4.8)
Serial interval –gamma (days): mean (95% CI)	7.4 (6.4–8.4)	9.3 (8–10.6)	10.9 (9.4–12.7)	12.9 (11.0–16.1)	18.3 (14.3–27.3)	11.4 (9.9–13.6)	2.9 (1.7–5.5)
Serial interval – lognormal (days): mean (95% CI)	7.5 (6.6–8.4)	9.1 (7.8–10.3)	8.9 (7.4–10.9)	12.9 (10.7–17.2)	22.0 (14.6–42.6)	11.6 (9.8–15.2)	4.2 (1.8–11)

*For each estimate, and for each accepted sample of the joint posterior parameter distribution, we computed the 2.5%, 25%, 50%, 75%, 97.5% quantiles as well as the mean and SD; the table reports the mean values and 95% CI of each quantity computed across the samples of the joint posterior distribution. Cells corresponding to the baseline distribution are highlighted in yellow.

Appendix Table 12. Estimated parameters for the Weibull, gamma and log-normal distributions of the serial interval for the two considered datasets after accounting for double censoring interval due to the discretization of dates.

Distribution	Dataset 1<br>Weibull	gamma	log-normal	Dataset 2<br>Weibull	gamma	log-normal
Offset (days)	1 (fixed)	1 (fixed)	1 (fixed)	6 (fixed)	6 (fixed)	6 (fixed)
Parameter 1: mean (95% CI)	Shape: 1.64 (1.15–2.13)	Shape: 1.95 (1.21–2.99)	Mean: 2.52 (2.22–2.82)	Shape: 2.21 (1.07–3.36)	Shape: 4.29 (1.73–9.20)	Mean: 1.52 (1.177–1.88)
Parameter 2: mean (95% CI)	Scale: 17.79 (13.68–21.9)	Scale: 8.03 (5.16–13.53)	SD: 0.89 (0.69–1.22)	Scale: 5.76 (3.88–7.64)	Scale: 1.17 (0.54–3.11)	SD: 0.52 (0.34–0.94)

Appendix Table 13. Summary statistics for different estimates of the serial interval after accounting for double censoring interval due to the discretization of dates*.

	Percentile				
Dataset 1	2.5%	25%	50%	75%	97.5%
Serial interval – Weibull (days): mean (95%CI)	2.7 (1.7–4.2)	9.0 (6.1–12.0)	14.9 (11.5–18.3)	22.7 (18.3–28.6)	41.1 (32.8–56.9)
Serial interval – gamma (days): mean (95%CI)	2.8 (1.6–4.4)	8.5 (5.8–11.3)	14.2 (11.0–17.8)	22.1 (17.7–28.6)	45.1 (35.1- 63.5)
Serial interval – lognormal (days): mean (95%CI)	3.1 (2.1–4.6)	7.7 (5.5–10.2)	13.3 (9.7–17.9)	23.2 (17.2–34.5)	69.3 (44.8–134.9)
Dataset 2					
Serial interval – Weibull (days): mean (95%CI)	6.9 (6.2–8.1)	9.0 (7.4–11.7)	10.7 (9.0–12.4)	12.5 (10.8–15.1)	16.8 (14.1- 24.3)
Serial interval –gamma (days): mean (95%CI)	7.5 (6.5–8.6)	9.3 (8.0–10.5)	10.7 (9.3–12.3)	12.3 (10.9–15.2)	16.7 (14.0–24.0)
Serial interval – lognormal (days): mean (95%CI)	7.6 (6.6–8.6)	9.2 (8.0–10.5)	10.5 (9.2–12.4)	12.4 (10.7–16.9)	18.4 (14.2–33.5)

*For each estimate, and for each accepted sample of the joint posterior parameter distribution, we computed the 2.5%, 25%, 50%, 75%, 97.5%; the table reports the mean values and 95% CI of each quantity computed across the samples of the joint posterior distribution.

Generation time

The generation time is defined as the difference between the date of infection of an infector and those of their infectees. We estimated two generation time distributions, one for dataset 1 and one for dataset 2, based on the same infector-infectee transmission pairs considered for the serial interval (Appendix Tables 7 and 8 respectively). As a baseline, we assumed that the generation time is gamma-distributed. For each dataset, we estimated the posterior distributions of the offset (θ), the shape (k) and scale (λ) parameters of a gamma distribution, based on an MCMC procedure with Metropolis-Hastings sampling, where the dates of exposure of each individual are considered as dummy parameters and the incubation period associated with them is included in the definition of the likelihood function (*2*). In the main analysis, exposure dates for the infectees are sampled between the date of exposure of the infector and the date of symptom onset of the infectee (i.e., a null prior is assumed for dates outside this interval). Under this assumption, the sampled date of exposure of infectees may precede the date of symptom onset of their infector, representing a potential pre-symptomatic transmission event. Exposure dates for infectors who are index case of the transmission chain are sampled with the only constraint that they need to occur before their symptom onset.

Convergence of MCMC trace plots was evaluated visually (see Appendix Figures 7 and 8). The MCMC procedure was run also for two other families of distributions (Weibull and log-normal). Posterior distributions of all estimated parameters are reported in Appendix Table 14. The 2.5%, 25%, 50%, 75%, 97.5% quantiles as well as the mean and standard deviation for each fitted distribution are reported in Appendix Table 15.

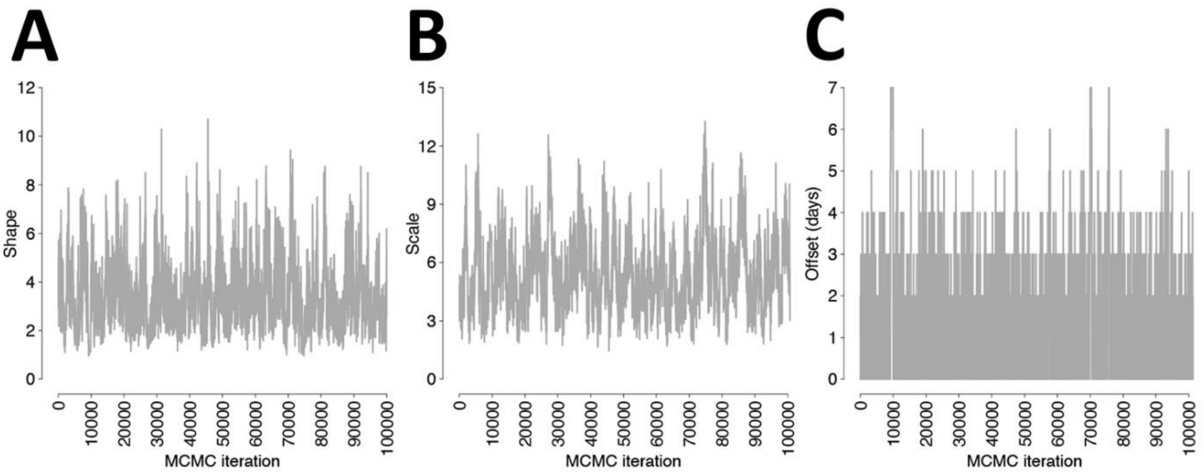

Appendix Figure 7. MCMC trace plots for the parameters of the gamma distribution of the generation time estimated from dataset 1. A) Shape parameter. B) Scale parameter. C) Offset.

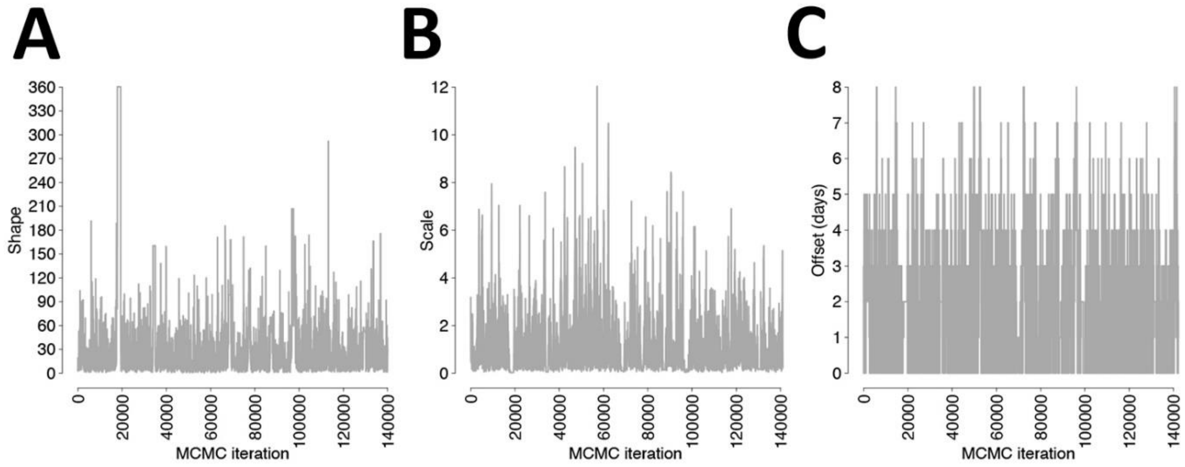

Appendix Figure 8. MCMC trace plots for the parameters of the gamma distribution of the generation time estimated from dataset 2. A) Shape parameter. B) Scale parameter. C) Offset.

Appendix Table 14. Estimated parameters for the Weibull, gamma and log-normal distributions of the generation time for the two considered datasets, considering the possibility of pre-symptomatic transmission*.

	Dataset 1			Dataset 2		
Distribution	Weibull	gamma	log-normal	Weibull	gamma	log-normal
Offset (days): median (95% CI)	1 (0–4)	1 (0–5)	1 (0–4)	2 (0–6)	1 (0–6)	3 (0–7)
Parameter 1: mean (95% CI)	Shape: 2.16 (1.43–3.15)	Shape: 3.42 (1.48–6.64)	Mean: 2.59 (2.22–2.86)	Shape: 2.17 (1.29–3.19)	Shape: 28.74 (2.03–166.05)	Mean: 2.00 (1.32–2.43)
Parameter 2: mean (95% CI)	Scale: 17.64 (13.85–21.68)	Scale: 5.25 (2.42–9.62)	SD: 0.56 (0.36–0.86)	Scale: 6.10 (4.38–8.38)	Scale: 1.01 (0.05–4.27)	SD: 0.22 (0.07–0.47)
Pre-symptomatic transmission (mean % and 95% CI)	20.3% (12.5%–28.1%)	20.4% (12.5%–28.1%)	19.3% (12.5%–28.1%)	16.7% (0.0%–54.5%)	17.4% (0.0%–54.5%)	15.4% (0.0%–54.5%)

*Cells corresponding to the baseline distribution are highlighted in yellow.

Appendix Table 15. Summary statistics for different estimates of the generation time, considering the possibility of pre-symptomatic transmission*

	Percentile						
Dataset 1	2.5%	25%	50%	75%	97.5%	mean	SD
Generation time –Weibull (days): mean (95%CI)	4.4 (2.0–7.1)	11.0 (7.8–14.1)	16.0 (12.8–19.2)	21.8 (18.5–26.0)	34.3 (27.5–44.9)	16.8 (14.1–19.9)	7.9 (5.7–11.1)
Generation time – gamma (days): mean (95%CI)	4.9 (2.2–7.6)	10.6 (7.5–13.6)	15.5 (12.3–18.9)	21.9 (18.0–27.0)	39.1 (29.5–52.4)	17.2 (14.1–20.9)	9.0 (6.0–12.9)
Generation time – lognormal (days): mean (95%CI)	6 (3.4–8.8)	10.6 (7.6–13.5)	14.8 11.6–18.1)	21.0 (16.9–26.6)	42.7 (29.5–68.1)	17.2 (13.9–21.7)	9.9 (5.7–18.2)
Dataset 2							
Generation time – Weibull (days): mean (95%CI)	7.3 (4.3–9.1)	9.8 (7.7–11.3)	11.0 (9.5–12.6)	12.1 (10.6–14.2)	14.0 (12.0–18.5)	10.9 (9.5–12.4)	1.7 (1.1–3.4)
Generation time –gamma (days): mean (95%CI)	6.9 (3.2–9.5)	9.3 (6.6–11.1)	10.9 (8.9–13.2)	12.9 (10.7–17.1)	17.5 (12.2–29.6)	11.3 (9.4–14)	2.7 (0.7–6.7)
Generation time – lognormal (days): mean (95%CI)	8.2 (5.3–10.1)	9.7 (7.7–11.2)	10.8 (9.3–12.2)	12.0 (10.5–14.4)	15.0 (11.5–22.9)	11.0 (9.6–12.6)	1.8 (0.5–4.4)

*For each estimate, and for each accepted sample of the joint posterior parameter distribution, we computed the 2.5%, 25%, 50%, 75%, 97.5% quantiles as well as the mean and standard deviation (SD); the table reports the mean values and 95% CI of each quantity computed across the samples of the joint posterior distribution. Cells corresponding to the baseline distribution are highlighted in yellow.

Sensitivity analysis without pre-symptomatic transmission

Since it is not known whether asymptomatic transmission is possible for MPXV clade I, we run a sensitivity analysis where we exposure dates for the infectee are sampled between the date of symptom of the infector (rather than the date of exposure of the infector) and the date of symptom onset of the infectee (i.e., a null prior is assumed for dates outside this interval). For this sensitivity analysis, we consider the baseline assumption of a gamma-distributed generation time. Posterior distributions obtained for the shape and scale parameters considering dataset 1 and 2 respectively, are reported in Appendix Table 16. The 2.5%, 25%, 50%, 75%, 97.5% quantiles as well as the mean and standard deviation for each fitted distribution are reported in Appendix Table 17. Estimates of the generation time under the assumption of no pre-symptomatic transmission are similar to the baseline analysis. The cumulative density functions of the estimated generation times are shown in Appendix Figure 9.

Appendix Table 16. Estimated parameters for the gamma distribution of the generation time for the two considered datasets, assuming that pre-symptomatic transmission is not allowed*

Parameter	Generation time (dataset 1)	Generation time (dataset 2)
Data source	Formenty et al. (*3*); Besombes et al. (*4*)	Learned et al. (*5*)
Distribution	Gamma	Gamma
Offset median (95%CI) (days)	1 (0–8)	3 (0–8)
Shape mean (95%CI)	5.57 (1.76–10.78)	40.73 (3.17 –138–97)
Scale mean (95% CI)	3.14 (1.49–6.32)	0.56 (0.07–2.43)

Appendix Table 17. Summary statistics for different estimates of the generation time, assuming that pre-symptomatic transmission is not allowed*

Dataset	Percentile					mean	SD
	2.5%	25%	50%	75%	97.5%		
Dataset 1							
Generation time – gamma (days): mean (95%CI)	7.1 (4.5–9.6)	12.2 (9.5–14.5)	16 (13.4–18.7)	20.9 (17.7–24.7)	32.9 (26.5–42.5)	17.1 (14.5–19.9)	6.7 (4.7–9.6)
Dataset 2							
Generation time –gamma, (days): mean (95%CI)	7.7 (4.7–9.6)	9.6 (7.7–11)	10.9 (9.5–12.5)	12.3 (10.7–15.2)	15.5 (11.9–23.3)	11.1 (9.7–13)	2.0 (0.8–4.7)

*For each estimate, and for each accepted sample of the joint posterior parameter distribution, we computed the 2.5%, 25%, 50%, 75%, 97.5% quantiles as well as the mean and SD; the table reports the mean values and 95% CI of each quantity computed across the samples of the joint posterior distribution.

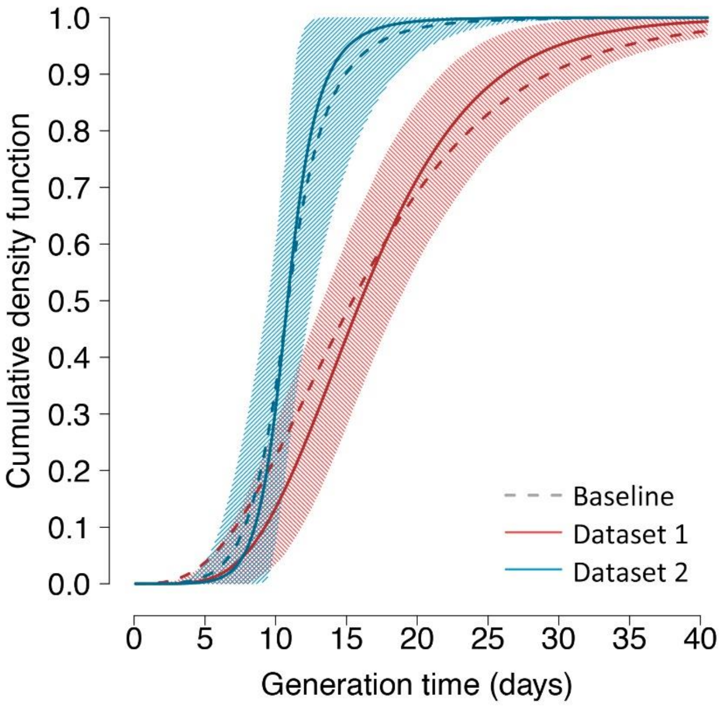

Appendix Figure 9. Cumulative density functions of the estimated generation times for the two considered datasets, when assuming that pre-symptomatic transmission is not allowed. The corresponding mean distributions obtained in the baseline analysis are reported with a dashed line for comparison.

Time-varying reproduction number, R_t

Temporal variations in the transmissibility of a pathogen can be monitored through the time-varying reproduction number, R_t, defined as the average number of secondary cases per infectious individual at time t. We estimate R_t by applying a commonly used statistical method

based on the renewal equation (*6*,*7*) and on the distribution of the generation time considering two different time-series:

- weekly laboratory-confirmed mpox cases in the Democratic Republic of the Congo (DRC) between week 1 and 19 of 2024 (from January 1 to May 12, 2024) (*8*);
- weekly hospitalized confirmed, probable or suspected mpox cases in the Kamituga Health Zone between week 39 of 2023 and week 16 of 2024 (from September 29, 2023, to April 21, 2024) (L.M. Masirika et al., unpub. data, https://www.medrxiv.org/content/10.1101/2024.05.10.24307057v1).

More specifically, the posterior distribution of R_t was estimated by applying MCMC to the following likelihood function:

$$\mathcal{L} = \prod_{t=1}^{T} P\left(C(t) - I(t);\ R_t \sum_{s=1}^{T} \varphi(s) C(t-s) \right)$$

Where:

- $P(k; \lambda)$ is the probability mass function of a Poisson distribution (i.e., the probability of observing k events if these events occur with rate λ).
- $C(t)$ is the total weekly number of new cases confirmed at week t;
- $I(t)$ is the total weekly number of new cases that are not locally transmitted (imported from another geographic setting or from the animal reservoir);
- R_t is the time-varying reproduction number at time t to be estimated;
- $\varphi(s)$ is the probability mass function of the generation time discretized by week, evaluated at week s.

For the estimation of R_t at the national level in the DRC, we considered zoonotic spillovers, by examining two possible importation scenarios:

- spillover 1: we assume that 50% (*9*; M.R. Ambrose et al., unpub. data, https://www.biorxiv.org/content/10.1101/677021v1) of weekly confirmed cases nationwide are due to zoonotic spillovers (i.e., they are considered as $I(t)$). The resulting epidemic curve under this assumption is shown in Appendix Figure 10A.

• spillover 2: we assume that 50% (*9*; M.R. Ambrose et al., unpub. data) of confirmed cases in the first 4 weeks of the considered period result from zoonotic spillovers. For the remaining part of the epidemic curve, we assume that the weekly number of zoonotic spillovers has an upper bound represented by the average of the first 4 weeks (Appendix Figure 10B).

Spillover scenario 1 represents a situation where the incidence of spillovers has changed over time (due, for example, to fluctuations in the rodent reservoir or in zoonotic contacts associated with habitat changes). According to spillover scenario 2, spillover incidence is approximately constant, and the change in the number of cases is driven by secondary transmission.

For what concerns the Kamituga Health Zone outbreak, phylogenetic analysis suggests that human mpox cases due to the newly detected clade Ib derive from a single introduction and are therefore attributable to sustained human to human transmission (*10*). For the estimation of R_t in Kamituga, we thus considered only one imported case at the beginning of the time series (Appendix Figure 11).

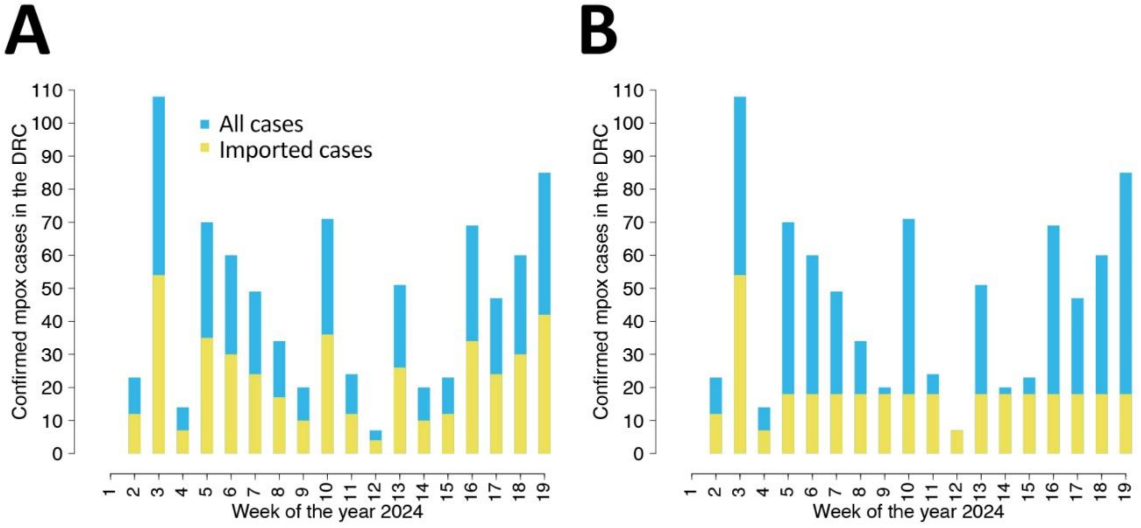

Appendix Figure 10. Time-series of laboratory-confirmed mpox cases in the Democratic Republic of the Congo by week of reporting (*8*), between week 1 and week 19 of 2024 (January 1–May 12, 2024). The weekly number of imported (spillover) cases was assumed according to spillover scenario; A) spillover 1: imported cases are 50% of the weekly lab-confirmed ones. B) spillover 2: imported cases are approximately constant over time, and equal to 50% of the weekly cases in the first 4 weeks.

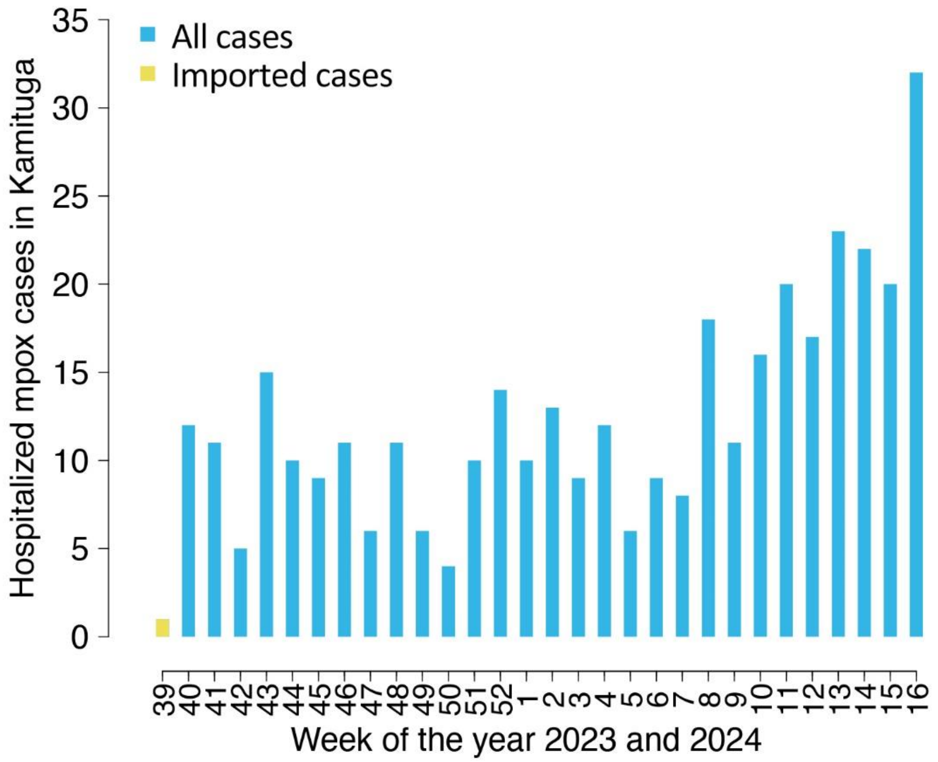

Appendix Figure 11. Time-series of hospitalized (confirmed, probable or suspected) mpox cases by week of reporting in the Kamituga Health Zone between week 39 of 2023 and week 16 of 2024 (September 29, 2023–April 21, 2024) (L.M. Masirika et al., unpub. data, https://www.medrxiv.org/content/10.1101/2024.05.10.24307057v1).

Estimates of R_t for the main analysis were computed considering the generation time distributions obtained both from dataset 1 and dataset 2 when allowing for pre-symptomatic transmission (R_t estimates for the DRC: Appendix Figure 12; R_t estimates for Kamituga: Figure 1 of the main text) or not (R_t estimates for the DRC: Appendix Figure 13; R_t estimates for Kamituga: Appendix Figure 14). The R_t values for the DRC oscillate around a mean of 0.60–0.83 in spillover scenario 1, and 0.70–0.95 in spillover scenario 2. These values are compatible with historical estimates of the reproduction number for MPXV clade I, ranging between 0.75 and 0.86 (*9*,*11*,*12*).

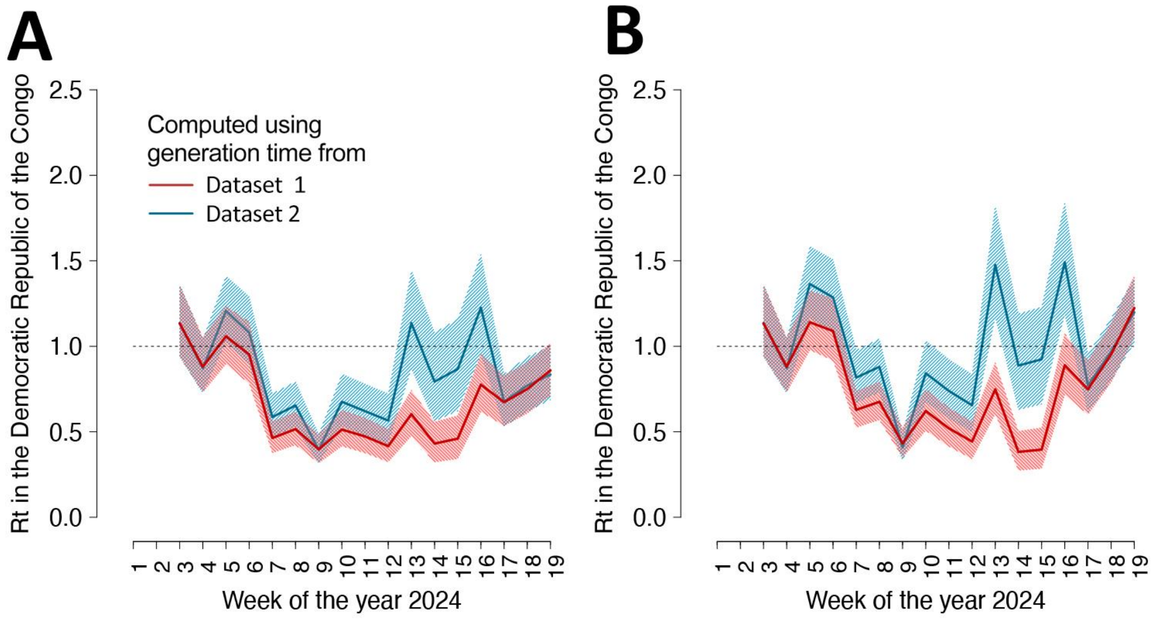

Appendix Figure 12. Estimates of the time-varying reproduction number (R_t) in the Democratic Republic of the Congo obtained from the time-series of reported cases, using the two estimates of the generation times (from dataset 1 and dataset 2), under the assumption of pre-symptomatic transmission (main analysis). A) spillover scenario 1; B) spillover scenario 2.

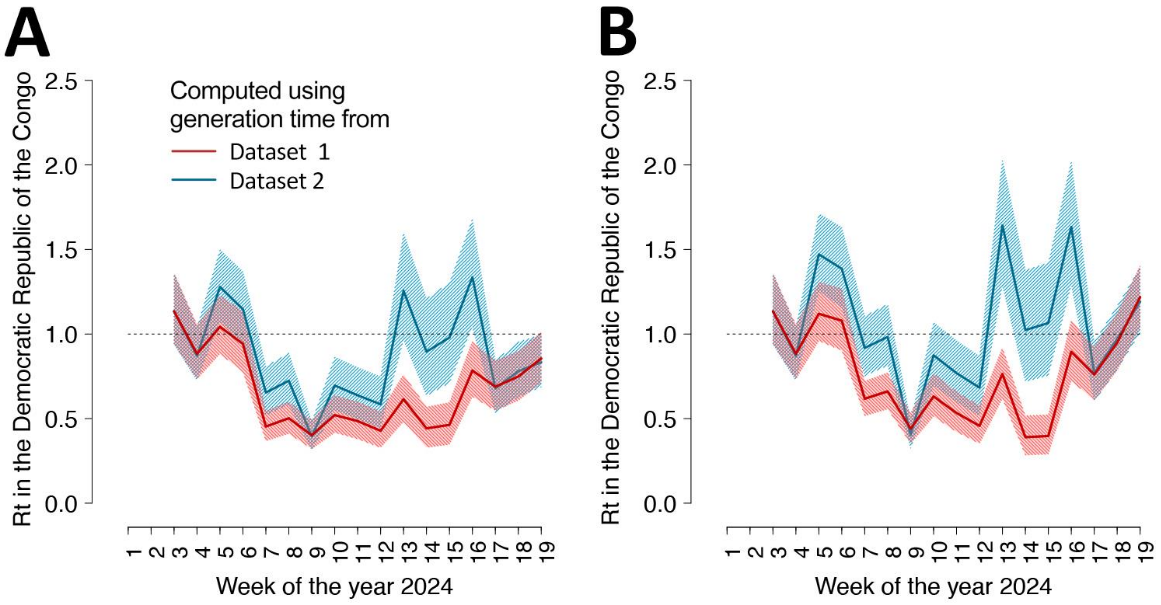

Appendix Figure 13. Estimates of the time-varying reproduction number (R_t) in the Democratic Republic of the Congo obtained from the time-series of reported cases, using the two estimates of the generation times (from dataset 1 and dataset 2), under the assumption of no pre-symptomatic transmission (sensitivity analysis). A) spillover scenario 1; B) spillover scenario 2.

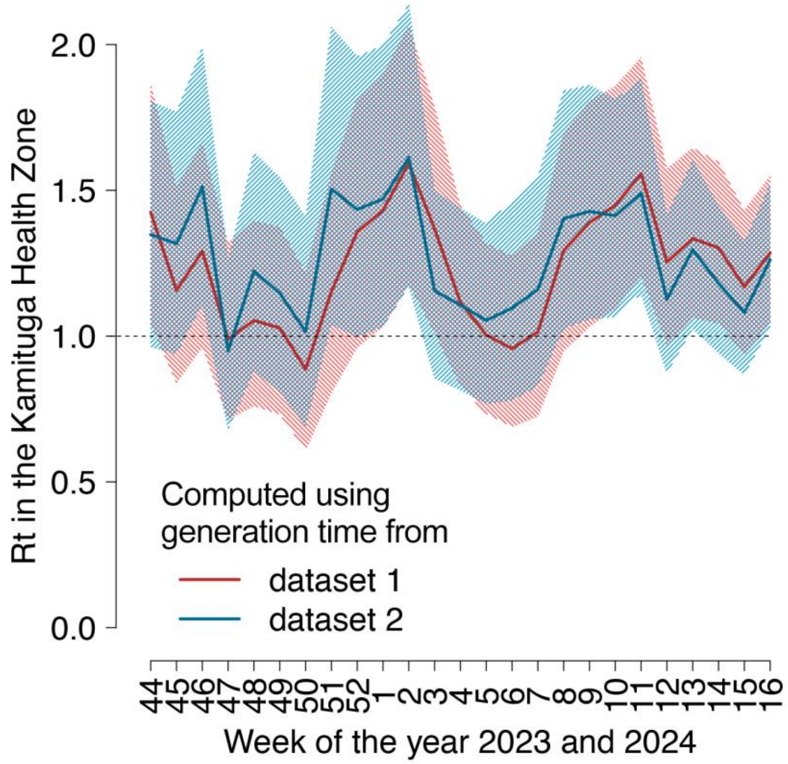

Appendix Figure 14. Estimates of the time-varying reproduction number (R_t) in the Kamituga Health Zone obtained from the time-series of reported cases, using the two estimates of the generation times (from dataset 1 and dataset 2), under the assumption of no pre-symptomatic transmission (sensitivity analysis).

Effective reproduction number, R_{eff}

We computed the effective reproduction number R_{eff} for the ongoing outbreak in Kamituga associated to a new subclade (Ib) of MPXV clade I (*10*), by using two alternative methods.

In the first method, we fitted a linear model to the logarithm of the weekly number of hospitalized cases, obtaining an exponential growth rate for the cases r = 0.067 (95%CI: 0.040–0.099). Then, we computed R_{eff} from the following equation (*13*):

$$R_{eff} = \int_0^\infty \frac{1}{\varphi(t)e^{-rt}}dt$$

where $\varphi(t)$ is the distribution of the generation time. Central estimates for the reproduction number R_0 were obtained by considering the mean value of r combined with

different estimates of the distribution of the generation time, while the uncertainty interval was computed by considering the boundaries of the 95%CI of the exponential growth rate r (see Appendix Table 18).

In the second method, we re-applied MCMC to the renewal equation above by constraining the value of the time-varying reproduction number to be fixed (*14*) ($R_t = R_{eff}$) between week 45 of 2023 and week 16 of 2024, a time window over which the time-varying reproduction number Rt remained approximately constant (Figure 1, main text). Mean and 95% CI of R_{eff} estimates obtained through the renewal equation are reported in Appendix Table 18 and are in good agreement with those resulting from the first method.

Appendix Table 18. Estimates of the reproduction number for the Kamituga outbreak*

Category	Generation time from dataset 1		Generation time from dataset 2	
	Exponential growth rate (mean and 95% UI)	Renewal equation (mean and 95% CI)	Exponential growth rate (mean and 95% UI)	Renewal equation (mean and 95% CI)
Pre-symptomatic transmission allowed (main analysis)	1.18 (1.10–1.26)	1.12 (1.0–1.24)	1.12 (1.07–1.17)	1.08 (0.97–1.21)
No pre-symptomatic transmission allowed (sensitivity analysis)	1.18 (1.10–1.27)	1.11 (0.99–1.24)	1.12 (1.07–1.17)	1.08 (0.96–1.2)

*CI, credible interval; UI, uncertainty interval.

References

1. Nolen LD, Osadebe L, Katomba J, Likofata J, Mukadi D, Monroe B, et al. Extended human-to-human transmission during a monkeypox outbreak in the Democratic Republic of the Congo. Emerg Infect Dis. 2016;22:1014–21. PubMed https://doi.org/10.3201/eid2206.150579

2. Guzzetta G, Mammone A, Ferraro F, Caraglia A, Rapiti A, Marziano V, et al. Early estimates of monkeypox incubation period, generation time, and reproduction number, Italy, May–June 2022. Emerg Infect Dis. 2022;28:2078–81. PubMed https://doi.org/10.3201/eid2810.221126

3. Formenty P, Muntasir MO, Damon I, Chowdhary V, Opoka ML, Monimart C, et al. Human monkeypox outbreak caused by novel virus belonging to Congo Basin clade, Sudan, 2005. Emerg Infect Dis. 2010;16:1539–45. PubMed https://doi.org/10.3201/eid1610.100713

4. Besombes C, Mbrenga F, Malaka C, Gonofio E, Schaeffer L, Konamna X, et al. Investigation of a mpox outbreak in Central African Republic, 2021-2022. One Health. 2023;16:100523. PubMed https://doi.org/10.1016/j.onehlt.2023.100523

5. Learned LA, Reynolds MG, Wassa DW, Li Y, Olson VA, Karem K, et al. Extended interhuman transmission of monkeypox in a hospital community in the Republic of the Congo, 2003. Am J Trop Med Hyg. 2005;73:428–34. PubMed https://doi.org/10.4269/ajtmh.2005.73.428

6. Wallinga J, Teunis P. Different epidemic curves for severe acute respiratory syndrome reveal similar impacts of control measures. Am J Epidemiol. 2004;160:509–16. PubMed https://doi.org/10.1093/aje/kwh255

7. Cori A, Ferguson NM, Fraser C, Cauchemez S. A new framework and software to estimate time-varying reproduction numbers during epidemics. Am J Epidemiol. 2013;178:1505–12. PubMed https://doi.org/10.1093/aje/kwt133

8. National Institute of Public Health. the Democratic Republic of the Congo. Monkeypox in the Democratic Republic of the Congo: epidemiological situation report no. 014 (06–12 May 2024) [in French] [cited 2024 Jun 25]. https://reliefweb.int/report/democratic-republic-congo/la-variole-simienne-monkeypox-en-republique-democratique-du-congo-rapport-de-la-situation-epidemiologique-sitrep-no014-06-12-mai-2024

9. Charniga K, McCollum AM, Hughes CM, Monroe B, Kabamba J, Lushima RS, et al. Updating reproduction number estimates for mpox in the Democratic Republic of Congo using surveillance data. Am J Trop Med Hyg. 2024;110:561–8. PubMed https://doi.org/10.4269/ajtmh.23-0215

10. Vakaniaki EH, Kacita C, Kinganda-Lusamaki E, O'Toole Á, Wawina-Bokalanga T, Mukadi-Bamuleka D, et al. Sustained human outbreak of a new MPXV clade I lineage in eastern Democratic Republic of the Congo. Nat Med. 2024 Jun 13 [Epub ahead of print]. PubMed https://doi.org/10.1038/s41591-024-03130-3

11. Fine PEM, Jezek Z, Grab B, Dixon H. The transmission potential of monkeypox virus in human populations. Int J Epidemiol. 1988;17:643–50. PubMed https://doi.org/10.1093/ije/17.3.643

12. Sun YQ, Chen JJ, Liu MC, Zhang YY, Wang T, Che TL, et al. Mapping global zoonotic niche and interregional transmission risk of monkeypox: a retrospective observational study. Global Health. 2023;19:58. PubMed https://doi.org/10.1186/s12992-023-00959-0

13. Wallinga J, Lipsitch M. How generation intervals shape the relationship between growth rates and reproductive numbers. Proc Biol Sci. 2007;274:599–604. PubMed https://doi.org/10.1098/rspb.2006.3754

14. Marziano V, Guzzetta G, Longini I, Merler S. Estimates of serial interval and reproduction number of Sudan Virus, Uganda, August–November 2022. Emerg Infect Dis. 2023;29:1429–32. PubMed https://doi.org/10.3201/eid2907.221718
